# Cycloartanes from *Oxyanthus pallidus* and derivatives with analgesic activities

**DOI:** 10.1186/s12906-016-1075-3

**Published:** 2016-03-09

**Authors:** Basile Nganmegne Piegang, Ignas Bertrand Nzedong Tigoufack, David Ngnokam, Angèle Sorel Achounna, Pierre Watcho, Wolfgang Greffrath, Rolf-Detlef Treede, Télesphore Benoît Nguelefack

**Affiliations:** Laboratory of Animal Physiology and Phytopharmacology, Department of Animal Biology, Faculty of Science, University of Dschang, P.O. Box 67, Dschang, Cameroon; Laboratory of Applied and Environmental Chemistry, Department of Chemistry, Faculty of Science, University of Dschang, P.O. Box 67, Dschang, Cameroon; Department of Neurophysiology, Centre for Biomedicine and Medical Technology Mannheim (CBTM), Heidelberg University, Ludolf-Krehl-Str. 13, D-68167 Mannheim, Germany

**Keywords:** Pain, Inflammation, *Oxyanthus pallidus*, Cycloartanes, Structure-activity relationship

## Abstract

**Background:**

The leaves of *Oxyanthus pallidus* Hiern (Rubiaceae) are extensively used in the west region of Cameroon as analgesic. These leaves are rich in cycloartanes, a subclass of triterpenes known to possess analgesic and anti-inflammatory properties. The present study aimed at evaluating the analgesic properties of three cycloartanes isolated from *Oxyanthus pallidus* leaves as well as their aglycones and acetylated derivatives.

**Methods:**

Three cycloartanes OP_3_, OP_5_ and OP_6_ obtained by successive chromatography of the crude methanol extract of the leaves were hydrolysed to yield respective aglycone AOP_1_, AOP_2_, AOP_3_ and acetylated to HOP_1_, HOP_2_ and HOP_3_ respectively. Formalin-induced pain model was used to evaluate the acute anti-nociceptive properties of these cycloartanes (5 mg/kg, *p.o*) in mice and to determine the structure-activity relationship. Acute (24 h) and chronic (10 days) anti-hyperalgesic and anti-inflammatory activities of OP_5_ were evaluated at the doses of 2.5 and 5 mg/kg/day administered orally. OP_6_ was also evaluated in acute experiments. The antioxidant and hepato-protective activities of OP_5_ were evaluated at the end of the chronic treatment.

**Results:**

The mixture and the individual isolated cycloartanes significantly inhibited both phases of formalin-induced pain with percentage inhibition ranging from 13 to 78 %. Acid hydrolysis did not significantly affect their antinociceptive activities while acetylation significantly reduced the effects of these compounds during the second phase of pain. OP_5_ and OP_6_ induced acute anti-hyperalgesic activity in formalin-induced mechanical hyperalgesia but not an anti-inflammatory effect. Repeated administration of OP_5_ for 10 days did not induce any anti-hyperalgesic effect. The evaluation of in vivo antioxidant properties showed that OP_5_ significantly reduced malondialdehyde and increased superoxide dismutase levels in liver without significantly affecting other oxidative stress and hepatotoxic parameters. Chronic administration of OP_5_ did not cause gastric ulceration.

**Conclusion:**

Cycloartanes isolated from *Oxyanthus pallidus* possess analgesic effects but lack anti-inflammatory activities. This analgesic effect especially on inflammatory pain may be due to the presence of hydroxyl group in front of the plane. OP_5_ is devoid of ulcerogenic effect and possess antioxidant properties that might be of benefit to its analgesic properties.

## Background

Pain is known as a major public health threat not only because it underlies almost every illness but also because it has become an illness by himself. It is therefore one of the leading causes of medical consultation [1], with enormous socio-economic and physiological deleterious impacts. Therefore, there is a high need to properly handle this ailment especially at its chronic stage. Huge progress has been made in recent years in order to first understand the pathology and secondly to master the therapeutic aspects and treatment. For the last purpose, a large number of therapeutics molecules like opioids, non-steroidal and steroidal analgesics have been developed. But existing therapeutics have some disadvantages such as their inefficacy, the resistance of some pathologies and the side effects, especially gastrointestinal, hepatic or renal toxicity. For instance, patients with severe inflammatory diseases respond poorly to conventional doses of corticosteroids [2, 3]. One of the exciting strategies to overcome these problems is to search for new and better drugs with minimal adverse effects. An important field of investigation to discover new therapeutic molecules that can treat more efficiently pain is that of secondary metabolites present in medicinal plants. In fact phytopharmacological research has brought substantial contribution to drug innovation. In addition, chemical structural modification of molecules has been shown to strongly modify the biological activity of initial compounds. In fact, the analgesic activity of a good number of chemical has been improved by their structural modification [4–6].

Triterpenes constitute a class of secondary metabolites that have shown potent analgesic and anti-inflammatory activities [7–9], one of the subclass being cycloartanes. The lasts molecules are secondary metabolites belonging to the class of tetracyclic triterpenes and are synthesized by many plant species. Cycloartanes possess various biological activities including anti-tumour, anti-virus, antibacterial, anti-inflammatory, analgesic or antioxidant activities [10, 11]. They therefore, offer an alternative in the treatment of pain.

Ayatollahi et al. [12] showed that cycloartanes are able to deactivate protein kinase C (PKC) and that this effect could be attributed to the presence of hydroxyl group in their molecular structures. Knowing the pivotal role that PKC plays in the transduction of pain [13, 14], we hypothesized that cycloartanes may possess good analgesic activity related to the presence of hydroxyl group.

The present study evaluates the analgesic and anti-inflammatory activities of three cycloartane glycosides, named pallidioside A (OP_3_), pallidioside B (OP_5_) and pallidioside C (OP_6_) isolated from the leaves of *Oxyanthus pallidus*, a plant extensively used in the West region of Cameroon as analgesic. In attempt to determine the importance of the glycoside moiety and the hydroxyl group in the biological activity of these compounds, the effects of their respective aglycone (AOP_1_, AOP_2_, AOP_3)_ and acetylated (HOP_1,_ HOP_2_, HOP_3_) derivatives were also evaluated.

## Methods

### Animal

Male and female rats of the Wistar strain aged 3 months and weighing between 180 and 200 g and Swiss mice of both sexes, aged 3 months and weighing between 20 and 30 g were used in this study. These animals were bred in the animal house of the Laboratory of Animal Physiology and Phytopharmacology of the University of Dschang under a 12-h light/dark cycle with free access to standard commercialized rodent diet and water. The number of animals used was the minimum possible to determine consistent effects of the drug treatments (See figures). All protocols were submitted and approved by the local Ethics Committee “The Animal Ethics Committee of the University of Dschang” and conformed to the guidelines for the study of pain in awake animals established by the International Association for the Study of Pain.

### Plant collection and extraction

#### Collection and isolation of major compounds

*Oxyanthus pallidus* materials were harvested in November in Dschang in the Western region of Cameroon. The plant was identified at the National Herbarium of Cameroon in Yaounde by Mr. Victor Nana, in comparison to an existing Voucher specimen n^o^ 7335/SFR/CAM. The leaves were subsequently dried at room temperature and then crushed. The resulting powder was extracted by soaking in methanol for 24 h. After filtration and evaporation at 70 °C under reduced pressure, the extract obtained was subsequently subjected to repeated column chromatography silica gel, which allowed to isolate and identify the three main compounds pallidiosides A (OP_3_), pallidiosides B (OP_5_) and pallidiosides C (OP_6_) as previously described [15].

#### Preparation of aglycone derivatives

One point five gram of each compound (OP_3_, OP_5_ and OP_6_) was individually refluxed in methanol/H_2_SO_4_ 10 % (25 ml) at room temperature for 48 h. Reaction mixture was neutralized by sodium hydroxide solution (6 N) and extracted with CHCl_3_ (3 × 10 ml). Each organic phase was concentrated under vacuum to give residues (560, 530 and 540 mg respectively) which were separately subjected to CC over silica gel with hexane/AcOEt (1:1) as the eluent to yield AOP_1_, AOP_2_ and AOP_3_ compounds (350, 335 and 340 mg respectively).

#### Preparation of acetylated derivatives

Two hundred and fifty milligrams of each compound (OP_3_, OP_5_ and OP_6_) were individually acetylated with Ac_2_O–pyridine (2:1) v/v at room temperature for 24 h, and each of the reaction mixture was purified over silica gel column, eluted with the hex-EtOAc (50 %) mixture to give the corresponding acetylated derivatives HOP_1_, HOP_2_ and HOP_3_ (270, 280 and 285 mg respectively).

### Pharmacological tests

#### Spontaneous pain induced by formalin in mice

This experiment followed the procedure previously described [16]. Animals were orally treated (gavage) with the mixture of the three cycloartanes (5, 10 and 20 mg/kg), individual isolated compounds (5 mg/kg) or derivatives (5 mg/kg). Diclofenac given orally at 5 mg/kg was used as positive control while the negative control group received 0.9 % NaCl solution. All the oral administered substances were given at equivalent volume of 0.5 ml/30 g body weight. One hour after administration of different substances, pain was induced by injecting 20 μL of formalin (2.5 % in saline) under the aponeurosis of the dorsal surface of the right hind paw of each animal. They were individually observed and pain evidenced by licking, flinching or biting the injected paw was quantified using an electronic timer, in two periods. The first period consisted of the first 5 min indicating neurogenic pain and second period (15th to 45th minute) corresponding to the tonic inflammatory pain.

#### Mechanical hyperalgesia and inflammation induced by formalin in rats

In an attempt to evaluate the time-dependent analgesic and anti-inflammatory effects, OP_5_ and OP_6_ were tested acutely in formalin-induced pain. The effect of 10 days repeated administration of OP_5_ (2.5 and 5 mg/kg/day as a single bolus) was also tested. NaCl 0.9 % and diclofenac were respectively used for negative and positive controls. One hour after oral administration of various substances, pain and inflammation were induced by injecting 0.1 ml of a 2 % formalin solution, under the left hind paw of each rat [17]. Ugo Basile analgesy meter no. 372157 and the caliper were used to measure the response threshold of each animal to mechanical pain and the paw diameter, respectively. The response threshold of the animal to mechanical pain was reported before treatment and 1, 2, 4, 6, 8 and 24 h post injection for the first day and once a day for ten days. The diameter of the injected paw was measured before treatment and 3, 5, 7 and 24 h post injection for the first day and then once a day for ten days. For repeated treatments, drugs were administered once a day, after collection of data. At the end of the experiment, the antioxidant, hepatotoxic and ulcerogenic effects of chronic administration of OP_5_ were examined. On this purpose, animals were sacrificed on day 11. Blood, kidney and liver samples were collected. Serum was separated for ALAT and ASAT assays. Kidney and liver were homogenized in Tris buffer and centrifuged. The supernatant was used to assay nitric oxide, malondialdehyde, superoxide dismutase, catalase and glutathione. Immediately after blood, liver and kidney collection, the stomach was carefully dissected out and opens along the great curvature. The mucus weight and ulceration index were estimated as previously described [18].

### Biochemical analysis

To assess whether different treatments could induce liver damage, enzymatic activities of aspartate aminotrasferase (AST) and alanine aminotrasferase (ALT) were measured in serum following the commercial kit (Immesco) procedure. SOD was evaluated based on its ability to inhibit or retard the autoxidation of adrenaline to adrenochrome in basic medium [19]. To assess whether the tested compound could inhibit lipid peroxidation, the amount of malondialdehyde was assayed in homogenates of liver and kidney. The protocol followed was as previously described by Ohkawa et al. [20]. Glutathione concentration and catalase activity in tissues homogenates were measured according to the methods described by Dimo et al. [21]. NO content in the homogenates of kidney and liver was measured, using Fiddler’s method [22].

### Statistical analysis

Results are expressed as Mean ± SEM (Standard Error of the Mean). One-way ANOVA followed by the Turkey’s post test was used to compare the averages for the various groups in the antinociceptive tests in mice and in biochemical analysis. Two-way ANOVA followed by Bonferroni post-test was used to compare different groups in the antihyperalgesic and the inflammatory tests. These analyses were performed with the aid of Graph Pad Prism software version 5.0.

## Results

### Cycloartanes

**Fig. 1:**
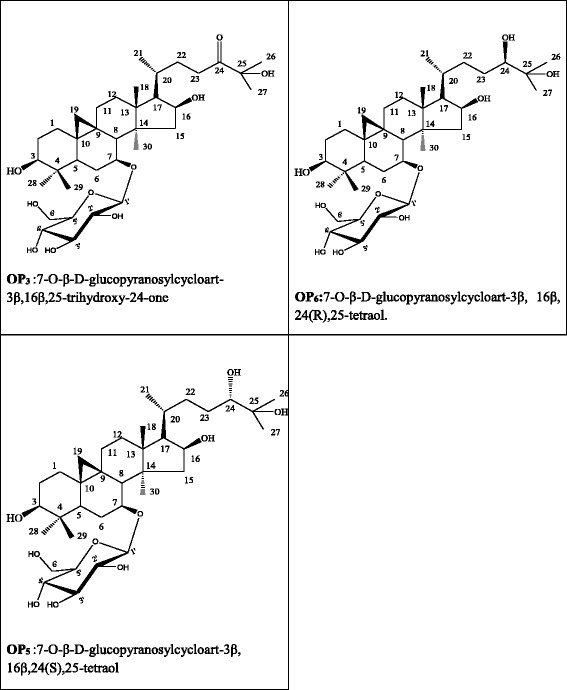
Structures of the main cycloartanes (pallidioside A: OP_3_, pallidioside B: OP_5_ and pallidioside C: OP_6_) isolates from the leaves of *Oxyanthus pallidus*

The individual main compounds are all characterized by the same basic structure and differ only on carbon 24. This carbon atom, bearing one *α*-OH in OP_5_ and one *β*-OH in OP_6_ respectively, was oxidized in ketone in molecule OP_3_ (Fig. 1). ^1^H and ^13^C data of these compounds were previously described by Tigoufack et al. [15].

**Fig. 2:**
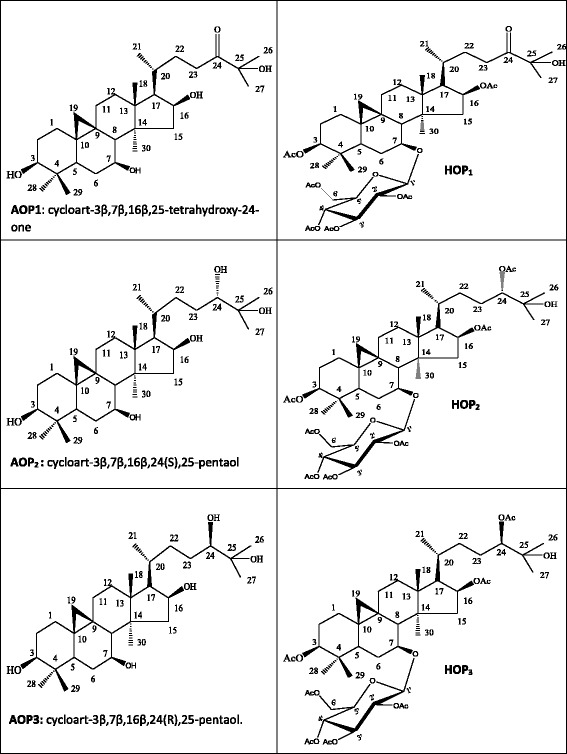
Structures of respective aglycones (AOP_1_, AOP_2_, AOP_3_) and acetylated (HOP_1_, HOP_2_ and HOP_3_) derivatives of OP_3_, OP_5_ and OP_6_

The aglycons derivatives generally have the same basic structure as the main compound from which they derived except the fact that, carbon atom C-7 which was linked to a glucosyl moiety in each precursor, was now linked to a *β*-OH group as indicated in Fig. 2.

AOP_1_ was obtained as a white powder. [α]D_23_ = + 85 (CH_3_OH, *c* = 0.92); IR (NaCl) ν_max_ (cm^−1^): 3375 (OH), 1703 (C = O), 1246 (C-O), ^1^H and ^13^C NMR data see Table 1. HR-TOFESIMS *m/z*: 513.3717 [M + Na]^+^ (calcd. For C_30_H_50_O_5_Na, 513.3712).

**Table 1:** ^1^H (600 MHz, MeOD) and ^13^C (150 MHz, MeOD) data of AOP_1_, AOP_2_ and AOP_3_

Positions	AOP_1_		AOP_2_		AOP_3_	
	δ_C_	δ_H_ *(mult, J(Hz)*	δ_C_	δ_H_ (*mult, J(Hz*)	δ_C_	δ_H_ (*mult, J (Hz)*
1	31.5	1.33 (1H, m, Hβ), 1.61 (1H, m, Hα)	31.3	1.33 (1H, m, Hβ), 1.60 (1H, m, Hα)	31.3	1.33 (1H, m, Hβ), 1.62 (1H, m, Hα)
2	30.5	1.64 (1H, m, Hα), 1.73 (1H, m, Hβ)	29.4	1.64 (1H, m, Hα), 1.73 (1H, m, Hβ)	29.4	1.63 (1H, m, Hα), 1.72 (1H, m, Hβ)
3	79.1	3.17 (dd, 4.6, 10.1)	77.7	3.24 (dd, 4.4, 11.1)	78.0	3.17 (dd, 4.5, 10.1)
4	41.2		39.8	/	39.8	/
5	45.6	1.51 (dd, 3.7, 13.1)	45.7	1.52 (dd, 3.9, 13.1)	45.7	1.51 (dd, 3.8, 12.1)
6	29.3	1.04 (1H, m, Hα), 1.74 (1H, m, Hβ)	30.7	1.04 (1H, m, Hα), 1.74 (1H, m, Hβ)	30.7	1.03, (1H, m, Hα), 1.74 (1H, m, Hβ)
7	70.1	3.50 ddd (4,0, 7.1, 12.6)	70.0	3.50 ddd (4,0, 7.1, 12.7)	70.0	3.50 (ddd, 4.0, 9.4, 13.3)
8	54.4	1.72 (1H, d, 7.0)	54 .2	1.71 (1H, d, 6.0)	54.2	1.72 (1H, d, 6.0)
9	26.6		26.5	/	26.5	/
10	19.7		19.6	/	19.7	/
11	26.0	1.31 (1H, m, Hα), 1.90 (1H, m, Hβ)	26.0	1.31 (1H, m, Hα), 1.92 (1H, m, Hβ)	26.0	1.30 (1H, m, Hα), 1.92 (1H, m, Hβ)
12	32.8	1.61 (1H, m, Hα), 1.68 (1H, m, Hβ)	32.4	1.63 (1H, m, Hα), 1.69 (1H, m, Hβ)	32.3	1.62 (1H, m, Hα), 1.68 (1H, m, Hβ)
13	45.3		45.4	/	45.4	/
14	46.0		46.0	/	46.0	/
15	48.8	1.64 (1H, dd, 5.2, 7.5, Hβ), 2.25 (1H, dd, 5.2 7.5, Hα)	48.8	1.66 (1H, dd, 5.2, 7.6, Hβ), 2.25 (1H, dd, 5.2, 7.6, Hα)	48.8	1.67 (1H, dd, 5.2, 7.7, Hβ), 2.25 (1H, dd, 5.2, 7.7, Hα)
16	72.1	4.43 (1H, ddd, 5.2, 7.5, 7.8)	72.1	4.44 (1H, ddd, 5.2, 7.6, 7.9)	72.0	4.43 (1H, ddd, 5.2, 7.6, 7.9)
17	55.8	1.63 (1H, dd, 7.8, 12.3)	55.9	1.64 (1H, dd, 7.9, 12.4)	55.8	1.63 (1H, dd, 7.9, 12.4)
18	17.2	1.20 (3H, s)	17.3	1.22 (3H, s)	17.2	1.21 (3H, s)
19	27.9	0.19 (1H, d, 4.0, Hα), 0.87 (1H, d, 4.0, Hβ)	27.9	0.19 (1H, d, 4.0, Hα), 0.85 (1H, d, 4.0, Hβ)	27.9	0.75 (1H, d, 4.0, Hα), 0.86 (1H, d, 4.0, Hβ)
20	30.1	1.81 (1H, m)	28.4	1.92 (1H, m)	28.5	1.82 (1H, m)
21	17.5	0.99 (1H, d, 7.6)	17.6	0.99 (1H, d, 7.6)	17.5	1.00 (1H, d, 7.6)
22	31.1	1.26 (1H, m, Hα),, 1.82 (1H, m, Hβ)	32.3	1.24 (1H, m, Hα), 1.82 (1H, m, Hβ)	32.5	1.22(1H, m, Hα),, 1.92(1H, m, Hβ)
23	28.3	1.63 (1H, m, Hα), 1.70 (1H, m, Hβ)	27.0	1.63 (1H, m, Hα), 1.72 (1H, m, Hβ)	28.0	1.62 (1H, m, Hα), 1.72 (1H, m, Hβ)
24	218.9		77.0	3.40 (1H, dd, 5.1, 12.0)	79.3	3.25 (1H, dd, 4.9, 11..3)
25	77.8		72.4	/	72.4	/
26	26.8	1.19 (3H, s)	24.0	1.19 (3H, s)	24.3	1.19 (3H, s)
27	26.8	1.17 (3H, s)	23.9	1.17 (3H, s)	23.4	1.16 (3H, s)
28	26.0	0.98 (3H, s)	24.6	0.98 (3H, s)	24.6	0.98 (3H, s)
29	13.2	0.86 (3H, s)	13.1	0.84 (3H, s)	14.9	0.86 (3H, s)
30	18.5	0.97 (3H, s)	18.4	0.97 (3H, s)	18.4	0.97 (3H, s)

AOP_2_ was obtained as a white powder. [α]D_23_ = + 58.6 (CH_3_OH, *c* = 0.89); IR (NaCl) ν_max_ (cm^−1^): 3465 (OH), 1249 (C-O), ^1^H and ^13^C data, see Table 1; HR-TOFESIMS *m/z*: 515.3719 [M + Na]^+^ (calcd. For C_30_H_52_O_5_Na, 515.3712).

AOP_3_ was obtained as a white powder. [α]D_23_ = + 34.2 (CH_3_OH, *c* = 1.20); IR (NaCl) ν_max_ (cm^−1^): 3477 (OH), 1250 (C-O), ^1^H and ^13^C data, see Table 1; HR-TOFESIMS *m/z*: 515.3719 [M + Na]^+^ (calcd. For C_30_H_52_O_5_Na, 515.3712).

The acetylated derivatives generally have the same basic structure as the main compound from which they derived except the fact that all primary and secondary hydroxyl groups were acetylated as showed in Fig. 2.

HOP_1_ was obtained as a white powder. [α]D_23_ = + 18.2 (CH_3_OH, *c* = 0.482 × 10^−2^); IR (NaCl) ν_max_ (cm^−1^): 3450 (OH), 1249 (C-O), 1715 (C = O). ^1^H and ^13^C data, see Table 2.

**Table 2:** ^1^H (500 MHz, MeOD) and ^13^C (150 MHz, MeOD) data of HOP_1_, HOP_2_ and HOP_3_

Positions	HOP_1_		HOP_2_		HOP_3_	
	δ_C_	δ_H_ *(mult, J(Hz)*	δ_C_	δ_H_ (*mult, J(Hz*)	δ_C_	δ_H_ (*mult, J (Hz)*
1	30.4	1.40 (1H, m, Hβ), 1.60 (1H, m, Hα)	29.1	1.62 (1H, m, Hβ), 1.72 (1H, m, Hα)	29.1	1.62 (1H, m, Hβ), 1.72 (1H, m, Hα)
2	29.1	1.62 (1H, m, Hα), 1.73 (1H, m, Hβ)	27.0	1.43 (1H, m, Hα), 1.62 (1H, m, Hβ)	27.0	1.43 (1H, m, Hα), 1.62 (1H, m, Hβ)
3	77.8	3.22 (dd, 4.4, 11.1)	77.8	3.23 (dd, 4.2, 10.9)	77.8	3.23 (dd, 4.2, 10.9)
4	39.8	/	39.8	/	39.8	/
5	44.3	1.42 (dd, 3.9, 13.3)	44.2	1.45 (dd, 3.9, 13.1)	44.2	1.45 (dd, 3.9, 13.1)
6	28.1	1.08 (1H, m, Hα), 2.13 (1H, m, Hβ)	28.1	1.10 (1H, m, Hα), 2.13 (1H, m, Hβ)	28.1	1.10 (1H, m, Hα), 2.13 (1H, m, Hβ)
7	80.0	3.63 (ddd (4,0, 7.63, 12.6)	80.0	3.63 (ddd (4,0, 7.1, 12.7)	80.0	3.63 (ddd (4,0, 7.1, 12.7)
8	50.2	2.09 (1H, d, 7.63)	50.0	2.11 (1H, d, 6.0)	50.0	2.11 (1H, d, 6.0)
9	20.1	/	20.2	/	20.2	/
10	26.4	/	26.4	/	26.4	/
11	26.5	1.33 (1H, m, Hα), 1.90 (1H, m, Hβ)	30.3	1.42 (1H, m, Hα), 1.62 (1H, m, Hβ)	30.3	1.42 (1H, m, Hα), 1.62 (1H, m, Hβ)
12	32.3	1.60 (1H, m, Hα), 1.68 (1H, m, Hβ)	32.4	1.60 (1H, m, Hα), 1.69 (1H, m, Hβ)	32.4	1.60 (1H, m, Hα), 1.69 (1H, m, Hβ)
13	46.4	/	46.4	/	46.4	/
14	45.3	/	45.3	/	45.3	/
15	46.6	1.64 (1H, dd, 5.0, 7.5, Hβ), 2.33 (1H, dd, 5.0 7.5, Hα)	46.4	1.63 (1H, dd, 5.2, 7.6, Hβ), 2.31 (1H, dd, 5.2, 7.6, Hα)	46.4	1.63 (1H, dd, 5.2, 7.6, Hβ), 2.31 (1H, dd, 5.2, 7.6, Hα)
16	71.9	4.46 (1H, ddd, 5.0, 7.6, 7.8)	72.3	4.43 (1H, ddd, 5.2, 7.6, 7.9)	72.3	4.43 (1H, ddd, 5.2, 7.6, 7.9)
17	56.0	1.62 (1H, dd, 7.7, 12.2)	56.0	1.64 (1H, dd, 7.9, 12.4)	56.0	1.64 (1H, dd, 7.9, 12.4)
18	16.2	1.18 (3H, s)	16.1	1.20 (3H, s)	16.1	1.20 (3H, s)
19	24.9	0.20 (1H, d, 4.5, Hα) 0.85 (1H, d, 4.5, Hβ)	24.7	0.18 (1H, d, 4.6, Hα), 0.87 (1H, d, 4.6, Hβ)	24.7	0.18 (1H, d, 4.6, Hα), 0.87 (1H, d, 4.6, Hβ)
20	29.7	1.72 (1H, m)	28.5	1.95 (1H, m)	28.5	1.91 (1H, m)
21	17.0	0.97 (1H, d, 7.6)	17.2	0.97 (1H, d, 7.6)	17.2	0.97 (1H, d, 7.6)
22	32.9	2.78 (1H, m, Hα), 2.86 (1H, m, Hβ)	32.4	1.22 (1H, m, Hα), 1.81 (1H, m, Hβ)	32.4	1.82 (1H, m, Hα), 1.01 (1H, m, Hβ)
23	29.5	1.21 (1H, m, Hα), 1.97 (1H, m, Hβ)	30.3	1.42 (1H, m, Hα), 1.62 (1H, m, Hβ)	31.3	2.12 (1H, m, Hα), 1.12 (1H, m, Hβ)
24	218.1	/	77.0	3.36 (1H, dd, 5.1, 12.0)	79.5	3.46 (1H, dd, 5.1, 12.0)
25	76.5	/	71.9	/	71.9	/
26	25.4	1.32 (3H, s)	25.4	1.32 (3H, s)	25.4	1.32 (3H, s)
27	24.8	1.00 (3H, s)	24.0	1.18 (3H, s)	24.5	1.18 (3H, s)
28	24.9	1.00 (3H, s)	24.9	1.00 (3H, s)	24.9	1.00 (3H, s)
29	12.2	0.82 (3H, s)	12.8	0.82 (3H, s)	12.8	0.82 (3H, s)
30	18.5	0.96 (3H, s)	18.5	0.94 (3H, s)	18.5	0.94 (3H, s)
1’	102.6	4.36 (1H, d, 7.8)	102.6	4.35 (1H, d, 7.7)	102.5	4.35 (1H, d, 7.7)
2’	73.9	3.18 (1H, dd, 7.8, 8.8)	73.9	3.18 (1H, dd, 7.7, 8.7)	73.9	3.18 (1H, dd, 7.7, 8.7)
3’	76.9	3.37 (1H, dd, 8.5, 8.8)	76.9	3.40 (1H, dd, 8.7, 9.0)	76.9	3.40 (1H, dd, 8.7, 9.0)
4’	70.2	3.28 (1H, dd, 8.5, 9.0)	70.2	3.27 (1H, dd, 9.0, 9.2)	70.2	3.27 (1H, dd, 9.0, 9.2)
5’	73.7	3.48 (1H, m)	73.7	3.48 (1H, m)	73.7	3.48 (1H, m)
6’	63.9	4.11 (1H, dd, 2.1, 11.9), 4.48 (1H, dd, 2.1, 11.9)	63.9	4.11 (1H, dd, 2.0, 12.0), 4.48 (1H, dd, 2.0, 12)	63.9	4.11 (1H, dd, 2.0, 12.0), 4.48 (1H, dd, 2.0, 12)

HOP_2_ was obtained as a white powder. [α]D_23_ = + 34.2 (CH_3_OH, *c* = 0.487 × 10^−2^); IR (NaCl) ν_max_ (cm^−1^): 3450 (OH), 1250 (C-O), 1713 (C = O). ^1^H and ^13^C data, see Table 2.

HOP_3_ was obtained as a white powder. [α]D_23_ = + 20.2 (CH_3_OH, *c* = 0.458 × 10^−2^); IR (NaCl) ν_max_ (cm^−1^): 3477 (OH), 1249 (C-O), 1718 (C = O). ^1^H and ^13^C data, see Table 2.

### Effect of the mixture and isolated cycloartanes on formalin-induced pain in mice

**Fig. 3:**
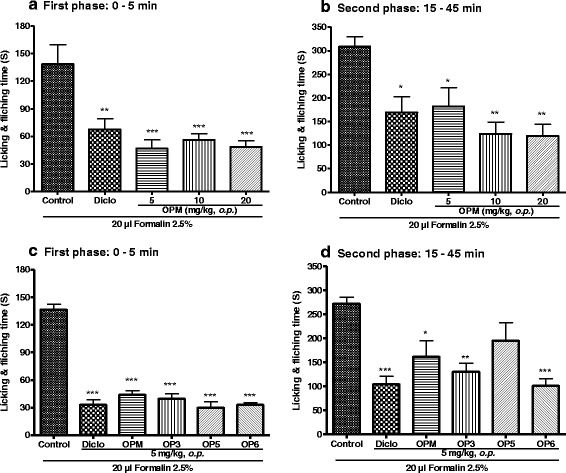
Effect of mixed (OPM) and isolated (pallidioside A: OP_3_, pallidioside B: OP_5_ and pallidioside C: OP_6_) cycloartanes on the first (panels **a** and **c**) and second (panels **b** and **d**) phases of formalin-induced pain in mice. Each bar represents the mean ± SEM, *n* = 6. *P < 0.05, **p < 0.01, ***p < 0.001 significant difference as compared to control group. Diclo: diclofenac

Treatment with different doses of the mixture of cycloartanes significantly reduced the pain behavior during the first and the second phases of pain induced by formalin as compared to the control group. The percentages of inhibition ranged from 51.33 to 65.15 % for the first phase (Fig. 3a) and from 45.15 to 61.42 % for the second phase (Fig. 3b). The highest percentages of inhibition were obtained at 5 mg/kg during the first phase and 20 mg/kg in the second. Concordantly, systemic oral administration of the three main compounds (OP_3_, OP_5_ and OP_6_) significantly (*p* <0.001) reduced the licking time of the paw during the first phase. No significant difference was observed between the effect of mixture and the individual compounds (Fig. 3c). OP_5_ reduced pain behavior by about 13 % during the second phase but this effect missed significance (*p* > 0.05), while OP_3_ and OP_6_ exhibited significant analgesic effect similar to that of diclofenac (Fig. 3d).

### Effect of OP_3_, its aglycone (AOP_1_) and acetylated (HOP_1_) derivatives on formalin-induced pain in mice

**Fig. 4:**
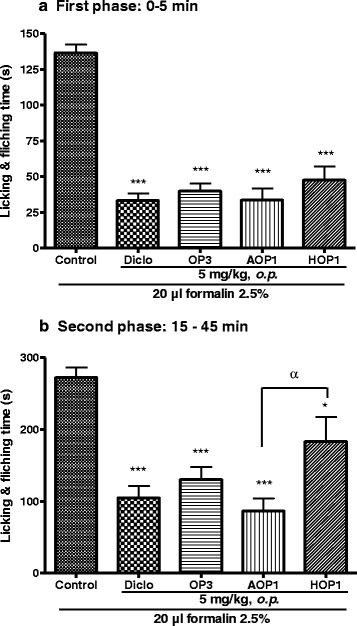
Effect of pallidioside A (OP_3_), its aglycone (AOP_1_) and acetylated (HOP_1_) derivatives on the first (panel **a**) and second (panel **b**) phases of formalin-induced pain in mice. Each bar represents the mean ± SEM of six animals. *p < 0.05, **p < 0.01, ***p < 0.001 significant different compared to the control. ^*α*^ *p < 0.05* significant difference between the two groups*.* Diclo: diclofenac

As shown in Fig. 4, oral administration of OP_3_ and its derivatives exhibited an effective analgesic effect on both phases of pain. There was no significant difference between the effect of OP_3_ and its derivatives during the first phase (Fig. 4a). At the second phase OP_3_ and derivatives also showed a significant analgesic effect but a significant difference was observed between the effect of the two OP_3_ derivatives, the aglycone AOP_1_ being significantly (*p* <0.05) more active than the acetylated HOP_1_ (Fig. 4b).

### Effect of OP_5_, its aglycone (AOP_2_) and acetylated (HOP_2_) derivatives on formalin-induced pain in mice

**Fig. 5:**
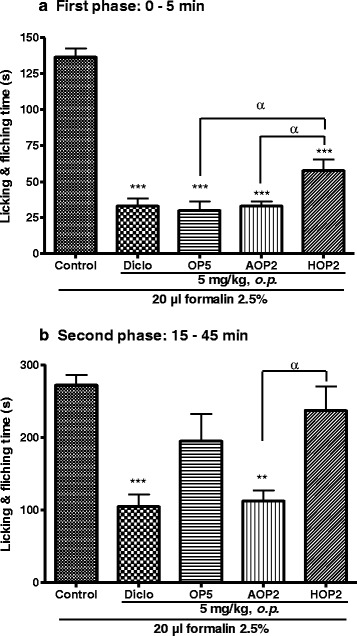
Effect of pallidioside B (OP_5_), its aglycone (AOP_2_) and acetylated (HOP_2_) derivatives on the first (panel **a**) and second (panel **b**) phases of formalin-induced pain in mice. Each bar represents the mean ± SEM, *n* = 6. **P <0.01, ***p <0.001 significant difference compared to control. ^*α*^ *p < 0.05* significant difference between the two groups*.* Diclo: diclofenac

Although OP_5_ and its derivatives all induced a significant analgesic effect on the first phase of pain induced by intraplantar administration of formalin, the acetylated derivative was significantly less potent than OP_5_ and its aglycone (Fig. 5a). On the second phase, OP_5_ and its acetylated derivative did not show significant analgesic effect as compared to control but AOP_2_ was significantly active as compared both to the control and HOP_2_ treated groups (Fig. 5b).

### Effect of OP_6,_ its aglycone (AOP_3_) and acetylated (HOP_3_) derivatives on formalin-induced pain in mice

**Fig. 6:**
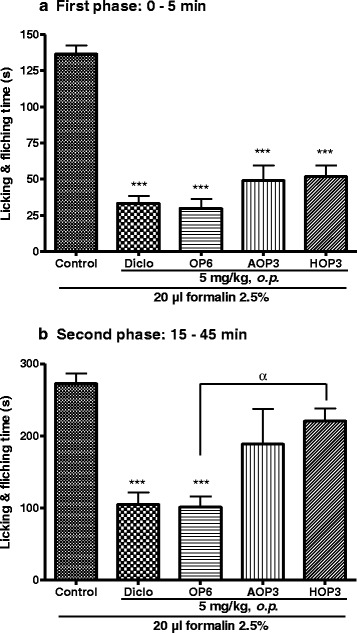
Effect of pallidioside C (OP_6_), its aglycone (AOP_3_) and acetylated (HOP_3_) derivatives on the first (panel **a**) and second (panel **b**) phases of formalin-induced pain in mice. Each bar represents the mean ± SEM, *n* = 6. ***p <0.001 significant differences compared to the control. ^*α*^ *p < 0.05* significant difference between the two groups. Diclo: diclofenac

OP_6_ and its two derivatives exhibited significant analgesic effect on the first phase of pain induced by formalin. Meanwhile, OP_6_ proved to be more effective than any of its derivatives (Fig. 6a). This was more marked at the second phase were AOP_3_ and HOP_3_ lacked significant activity. However, OP_6_ was significantly active as compared both to the control group and the HOP_3_ treated group (Fig. 6b).

### Effect of OP_5_ and OP_6_ on mechanical hyperalgesia and inflammation induced by formalin in rats

**Fig. 7:**
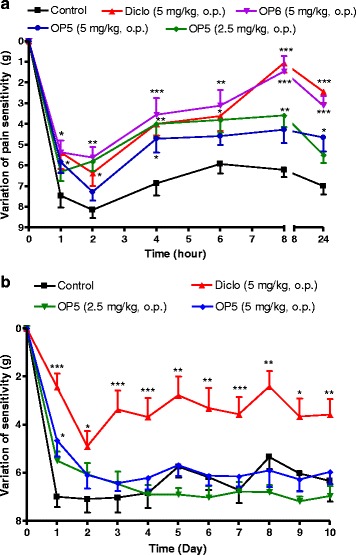
Time-dependant acute (panel **a**) effect of Pallidioside B (OP_5_) and Pallidioside C (OP_6_) and repeated treatment of OP_5_ (panel **b**) on mechanical pain induced by Randal sellito in rat paw injected with formalin (2 %, 100 μl). Animals were orally treated with different compounds and injected one hour later in the paw with formalin. Pain threshold was measured before treatment and after formalin injection. For chronic experiment, animals were daily treated and pain threshold was measured before drug administration. Each point represents the mean ± SEM, *n* = 8. *P < 0.05, **p < 0.01, ***p < 0.001 significant differences compared to the control. Diclo: diclofenac

Systemic oral administration of OP_5_ and OP_6_ one hour before induction of pain both produced significant inhibition of hyperalgesia for up to 24 h. The acute maximal effect was observed 8 h post induction with OP_6_ at the dose of 5 mg/kg as compared to the negative control (Fig. 7a). This effect was similar to that of diclofenac at the same dose. During the 10 days treatment, OP_5_ at both doses shows significant effect only at the first day (Fig. 7b).

**Fig. 8:**
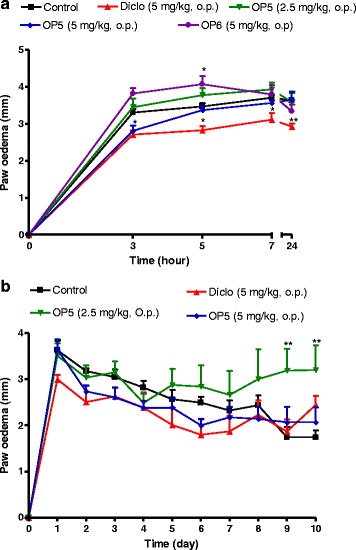
Time-dependant acute (panel **a**) effect of Pallidioside B (OP_5_) and Pallidioside C (OP_6_) and repeated treatment of OP_5_ (panel **b**) on inflammation induced by formalin (2 %, 100 μl) injection in rat paw. Animals were orally treated with different compounds and injected one hour later with formalin. The diameter of the paw was measured before treatment and after formalin injection. For chronic experiment, animals were daily treated and the diameter of the paw was measured before drug administration. Each point represents the mean ± SEM, *n* = 8. *P < 0.05, **p < 0.01, ***p < 0.001 significant differences compared to the control. Diclo: diclofenac

As shown in Fig. 8, OP_6_ instead increase the inflammation induced by formalin. The inflammation was significantly reduced by OP_5_ at the dose of 5 mg/kg at the third hour post induction. Repeated administration of OP_5_ at both doses did not show any anti-inflammatory activity all along the 10 days of experiment.

### Biochemical analyses

Results of the assays of oxidative stress parameters are presented in Table 3. After 10 days of repeated treatment, OP_5_ significantly reduced the malondialdehyde production and increased the superoxide dismutase activity in the liver, but has no effect on these parameters in the kidney. OP_5_ did not affect the concentration of NO, glutathione and the activity of catalase both in the liver and kidneys. The concentration of ALAT dose-dependently increased in the serum of OP_5_ treated animals as compared to the control group. Meanwhile, the effect was not statistically significant.

**Table 3:** Effects of different treatments on the biochemical parameters of oxidative stress and hepatotoxicity

Parameters	Tissue	Control	Diclofenac (5 mg/kg)	OP5 (2.5 mg/kg)	OP5 (5 mg/kg)
NO (μmol/g tissue)	liver	0.49 ± 0.06	0.28 ± 0.06*	0.48 ± 0.04	0.36 ± 0.03
	kidney	1.014 ± 0.11	0.66 ± 0.15	1.23 ± 0.15	0.85 ± 0.06
Glutathione (μmol/g tissue)	liver	0.12 ± 0.01	0.11 ± 0.01	0.12 ± 0.01	0.10 ± 0.01
	kidney	0.07 ± 0.01	0.04 ± 0.01	0.09 ± 0.01	0.07 ± 0.00
MDA (μmol/g tissue)	liver	9.37 ± 0.74	9.80 ± 0.46	3.10 ± 0.37***	9.42 ± 0.24
	kidney	0.12 ± 0.01	0.09 ± 0.02	0.14 ± 0.01	0.12 ± 0.00
SOD (U/mg protein)	liver	0.05 ± 0.01	0.12 ± 0.00*	0.08 ± 0.02	0.11 ± 0.01*
	kidney	0.19 ± 0.08	0.12 ± 0.05	0.09 ± 0.02	0.10 ± 0.04
Catalase (U/mg protein)	kidney	0.08 ± 0.01	0.08 ± 0.01	0.10 ± 0.02	0.08 ± 0.01
ALAT (U/mg protein)	serum	0.39 ± 0.15	0.4 ± 0.01	0.60 ± 0.08	0.80 ± 0.21
ASAT (U/mg protein)	serum	1.28 ± 0.10	1.58 ± 0.13	1.35 ± 0.07	1.313 ± 0.11

Each value represents the average ± ESM of eight animals.^*^ *p* < 0.05, ^***^ *p* < 0.001 significant difference compared to the control group

### Ulcerogenic activity

It is observed from Table 4 that after 10 days treatment, animals treated with OP_5_ at both doses showed no ulceration in their gastric mucosa. Ulceration was recorded in all animal treated with diclofenac with a percentage ulcerated surface of 15.95 %. It was also observed that repeated administration of diclofenac at the dose of 5 mg/kg/day significantly reduced the mucus production, whereas OP_5_ has no effect.

**Table 4:** Evaluation of the ulcerogenic activity of Diclofenac (Diclo) and OP_5_

Groups	Doses (mg/kg)	Mucus weight (mg)	Total surface of stomach (mm^2^)	ulcerated surface (mm^2^)	% of ulcerated animals
Control		0.163 ± 0.013	1479.00 ± 72.79	0.00 ± 0.00	0
Diclo	5	0.073 ± 0.014*	1406.00 ± 78.92	221.10 ± 0.00***	100
OP5	2,5	0.127 ± 0.018	1886.00 ± 127.00	0.00 ± 0.00	0
OP5	5	0.090 ± 0.009	1723.00 ± 46.90	0.00 ± 0.00	0

Each value represents the mean ± SEM of eight animals.*p < 0.05, ***p < 0.001 significant difference compared to the control group. *Diclo* diclofenac

## Discussion

This work was undertaken to evaluate the analgesic and anti-inflammatory effects of three cycloartanes isolated from *Oxyanthus pallidus* and their derivatives. The mixture of cycloartanes significantly inhibited both phases of pain induced by the injection of formalin. When tested individually, isolated compounds exhibited different intensities of activity. During the first phase of pain, the three compounds showed significant antinociceptive effect. No significant difference was observed between their effects although OP_5_ was nominally the most effective. Regarding the second phase of pain, OP_3_ and OP_6_ induced a significant analgesic effect while OP_5_ showed less activity. It is well known that formalin model which is consider mimicking clinical pain [23], has two main distinct phases. The first one characterized as neurogenic pain is related to chemical stimulation of A_δ_ and C fibers, the release of excitatory amino-acids, nitric oxide and substance P while the second phase known as inflammatory pain is related to the release of bradykinin and prostaglandins [16, 24]. Drugs that inhibit only the first or both phases are considered as central analgesics while those acting only at the second phase are considered as peripheral analgesic drugs [25]. The mixture of cycloartanes as well as OP_3_ and OP_6_ significantly inhibited both phases of formalin-induced pain while OP_5_ inhibited predominately the first phase. The activity of these compounds either taken individually or as a mixture was significantly more potent at the first phase than at the second. These results indicate that the tested cycloartanes may be central acting molecules. The structural difference between these compounds is at C-24, which in the case of OP_3_ carries a ketone while that of OP_5_ and OP_6_ carry a hydroxyl group. The combination of the structure and activity of molecules shows that the substitution on C-24 by the ketone group or the hydroxyl group does not significantly affect the analgesic activity in the formalin model. However, the analgesic activity on the inflammatory pain can be improved if the hydroxyl group at C-24 is placed in front of the plane.

Acid hydrolysis leading to aglycone did not affect the analgesic activity of different compounds on the neurogenic pain but significantly reduced the effect of OP_6_ on the inflammatory pain. These results suggest a probable interaction between the hydroxyl group and sugar, all placed in front of the plane, which could be necessary for the activity of OP_6_. This hypothesis is further supported by the results obtained after acetylating OP_3_, OP_5_ and OP_6_. In fact, the process has significantly inhibited the analgesic activity of all these compounds. We can then conclude that the hydroxyl groups attached to the aglycone are responsible for the analgesic activity of these cycloartanes, mainly as far as the inflammatory pain is concern. These results corroborate those previously obtained by Ayatollahi et al. [12] who showed that cycloartanes can deactivate protein Kinase C, thanks to their hydroxyl group. In addition, it has been shown that PKC mediates the phase 2 of formalin-induced pain [26]. Taking all together, it can be thought that cycloartanes from *Oxyanthus pallidus* induced their analgesic effect by inhibiting at least partially the activation of PKC.

OP_5_ and OP_6_ were further tested on mechanical hyperalgesia and inflammation induced by formalin in rats. In the acute test, they significantly reduced mechanical hyperalgesia from the first hour after induction of pain and lasted until the 24th hour post administration. OP_5_, in contrary of OP_6_, showed a significant anti-inflammatory effect only at the 3rd hour post treatment. These results indicate that the analgesic effects of tested cycloartanes are not dependent to their anti-inflammatory activity if any. Furthermore, repeated treatment with OP_5_ did not show any anti-inflammatory effect, demonstrating that OP_5_ is devoid of anti-inflammatory effect.

It was observed that unlike diclofenac, repeated oral administration of OP_5_ did not induce gastric ulceration and did not significantly affect the gastric mucus weight. Diclofenac reduces mucus production and induces gastric ulcer through the reduction of prostaglandin resulting from its inhibitory effect on cyclooxygenase. The above result suggests that OP_5_ do not inhibit cyclooxygenase.

The strong involvement of oxidative stress in inflammatory and painful phenomena [27, 28] has motivated the study of the in vivo antioxidant activity of OP_5_ in animals treated chronically. OP_5_ significantly reduced malondialdehyde (MDA) and increased the level of superoxide dismutase (SOD) without affecting the activity of catalase and nitric oxide amount. These results suggest that OP_5_ might potentiate the in vivo antioxidant capacity of the organism by increasing the SOD activity that could lead to reduction of lipid peroxydation. In other to evaluate the impact of OP_5_ on the liver integrity, alanine aminotrasferase (ALAT) and aspartate aminotrasferase (ASAT) activities were assayed in the serum. A dose dependent increase in ALAT was observed in OP_5_ treated group, although not significant. It is well known that plasma increase in ALAT and ASAT, especially ALAT is a result of liver cells necrosis. Thus, even though there was no evidence of hepatotoxic effect, caution should be taken when using OP_5_.

## Conclusion

Cycloartanes isolated from *Oxyanthus pallidus* possess analgesic effects that are dissociated from anti-inflammatory activity if any. The analgesic effect of these compounds especially the effect on inflammatory pain may be due to the presence of hydroxyl group in front of the plane. Meanwhile, these cycloartanes are more potent on the neurogenic than on the inflammatory pain.
